# A Survey on Multi-Sensor Fusion Perimeter Intrusion Detection in High-Speed Railways

**DOI:** 10.3390/s24175463

**Published:** 2024-08-23

**Authors:** Tianyun Shi, Pengyue Guo, Rui Wang, Zhen Ma, Wanpeng Zhang, Wentao Li, Huijin Fu, Hao Hu

**Affiliations:** 1 China Academy of Railway Sciences, 2 Daliushu Road, Haidian District, Beijing 100081, China; 2 Institute of Electronic Computing Technology, China Academy of Railway Sciences, 2 Daliushu Road, Haidian District, Beijing 100081, China

**Keywords:** high-speed railways, multi-sensor fusion, perimeter intrusion, object detection

## Abstract

In recent years, the safety issues of high-speed railways have remained severe. The intrusion of personnel or obstacles into the perimeter has often occurred in the past, causing derailment or parking, especially in the case of bad weather such as fog, haze, rain, etc. According to previous research, it is difficult for a single sensor to meet the application needs of all scenario, all weather, and all time domains. Due to the complementary advantages of multi-sensor data such as images and point clouds, multi-sensor fusion detection technology for high-speed railway perimeter intrusion is becoming a research hotspot. To the best of our knowledge, there has been no review of research on multi-sensor fusion detection technology for high-speed railway perimeter intrusion. To make up for this deficiency and stimulate future research, this article first analyzes the situation of high-speed railway technical defense measures and summarizes the research status of single sensor detection. Secondly, based on the analysis of typical intrusion scenarios in high-speed railways, we introduce the research status of multi-sensor data fusion detection algorithms and data. Then, we discuss risk assessment of railway safety. Finally, the trends and challenges of multi-sensor fusion detection algorithms in the railway field are discussed. This provides effective theoretical support and technical guidance for high-speed rail perimeter intrusion monitoring.

## 1. Outline

**Figure 1:**
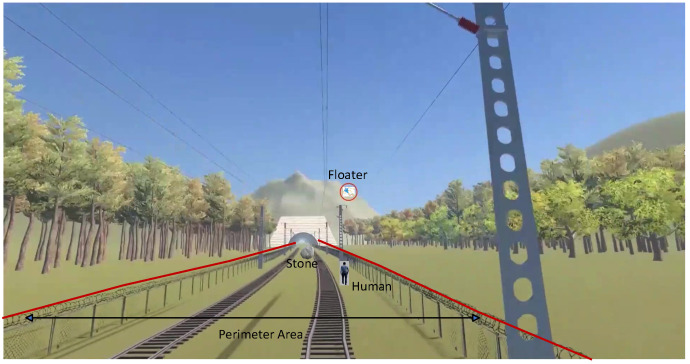
Perimeter intrusion.

According to the International Union of Railways [1], the total mileage of the world’s railways has reached 1.1 million kilometers, of which the total mileage of high-speed railways reached 59,000 km by the end of 2022. The complexity of terrain or the negligence or subjective malice of relevant actors can lead to the existence of uncontrollable risk points along a railway. The factors that affect the safety of railway systems are diverse, including locomotive parts [2], rail conditions [3], wireless networks [4], station management [5], and track safety [6]. This research focuses on track safety. The high-speed railway perimeter is an important protective area along the track, excluding the stations. The high-speed railway perimeter [7] is the boundary of the high-speed railway line area that needs to be physically protected or electronically protected. Incidents such as personnel intrusion and rockfall intrusion occur frequently, seriously threatening the safety of high-speed railways. An example of perimeter intrusion is shown in Figure 1.

Most high-speed railway perimeters adopt closed models; however, it is difficult to fully cover all the risk points, and loopholes and blind spots can still exist within the perimeter. The large-scale delay of trains caused by perimeter intrusion seriously affects transportation efficiency and may even cause casualties and economic losses. Therefore, fast and accurate detection of high-speed railway perimeter intrusion is urgent and can save time for the disposal of trains. At present, all countries have taken protective measures for high-speed railway perimeter security. However, there are still false alarms and missed alarms due to the poor noise resistance. Consequently, it is necessary to summarize the prevention technologies for high-speed railway perimeters and analyze the advantages and disadvantages of each technical means. This will provide directions for future research.

**Figure 2:**
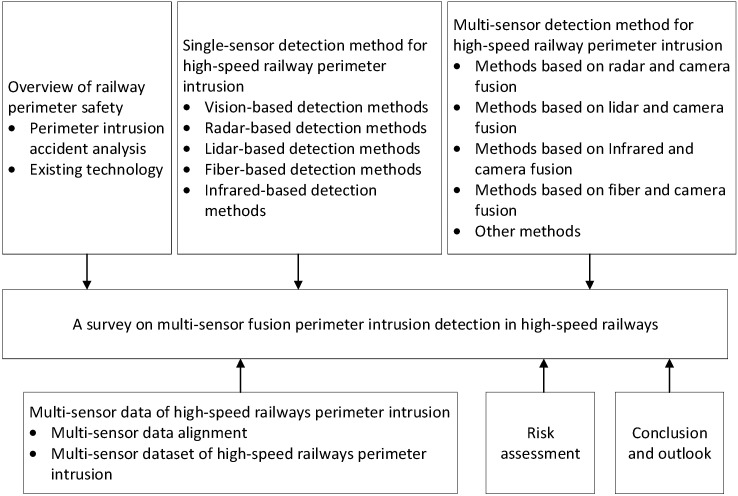
Paper structure.

In this paper, we first summarize the high-speed rail perimeter intrusion events. We analyze the risk points prone to perimeter intrusion and the current protection measures. Secondly, we discuss the types of sensors used for perimeter intrusion monitoring currently and the adaptability of single-sensor target recognition algorithms, including camera, lidar, radar, infrared, and fiber. Next, we study the perimeter intrusion multi-sensor detection and identification method, multi-sensor data alignment method, and multi-sensor dataset for the railway field by combining the advantages and disadvantages of each monitoring sensor. Then, we discuss risk assessment of railway safety. Finally, a summary is presented. Future research on multi-sensor perimeter intrusion detection and identification technology is proposed. The structure of this article is shown in Figure 2.

## 2. Overview of High-Speed Rail Perimeter Intrusion Security Issues

### 2.1. Perimeter Intrusion Event Analysis

**Figure 3:**
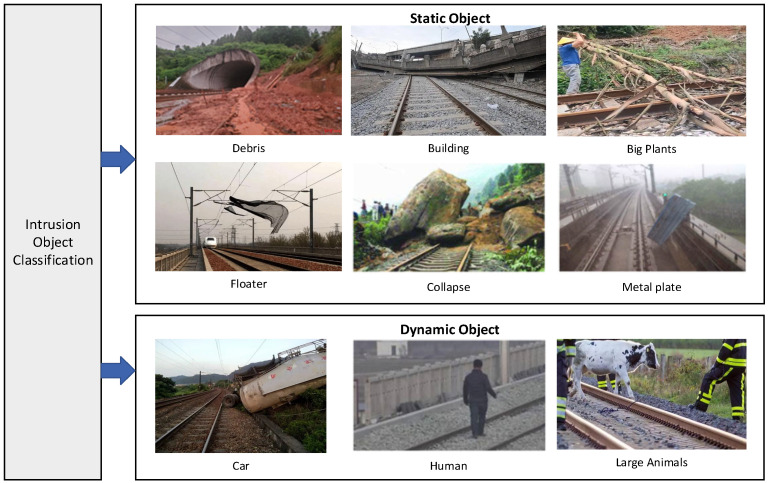
Intrusion object classification.

According to statistics [8], more than 83% of serious accidents on European railways are caused by perimeter intrusion. Nearly 1000 people die in such accidents on EU railways every year, with a total annual accident cost of around EUR 3.2 billion. In 2022, 954 total railway fatalities and 5882 injuries were reported by the National Safety Council [9], with 29 percent of those fatalities occurring at railway crossings. India’s railway lines are more than 68,000 km long, with more than 12,000 passenger trains and over 7000 freight trains daily. According to the annual report of Indian Railways [10], a total of 16,431 deaths and 1852 injuries were recorded in railway accidents in 2021, of which 67.7 percent were from falls from trains or collisions with people on the tracks. Statistics on traffic accidents on China’s high-speed railways show that perimeter intrusion accounts for about 79 percent of the incidents. Statistics show that about 55 percent of the incursions occur at the subgrade, followed by bridgeheads and tunnel entrances at about 35 percent. By summarizing and analyzing the domestic and international railway accident data, the intrusion targets [8,9,10] that endanger train operation are classified and counted, as shown in Figure 3.

In addition, vulnerable points of intrusion targets can be classified according to the subgrade, tunnels, and bridges. Among them, the subgrade is a frequent area of invasion due to its flat, open terrain and close proximity to human habitation or areas of dense vegetation. The main categories of incursions are large animals, vegetation, damaged buildings, humans, or vehicles. There are large areas of earthy hills and vegetation in the tunnel area, which are susceptible to vegetation intrusion, rockfall encroachment, landslide/collapse, and debris-flow caused by landslides. The main types of bridge intrusions are personnel intrusions, light floater intrusions, etc.

### 2.2. Existing Security Method

In Europe, the SMART (Smart Automation of Rail Transport) railway monitoring system was developed using on-board multi-sensor technology [11]. This system combines thermal imagers with laser scanners to create a fusion system that enables mid-range (up to 200 m) and remote (up to 1000 m) obstacle detection during the day and night. To avoid train accidents due to collision or obstacle intrusion, a [12] system consisting of two infrared sensors and an ultrasonic sensor was proposed for stopping a train in the case of an obstacle in front of it in India. The Japan Railway Corporation (JR-East) [13] designed obstacle intrusion detection systems at level crossings to prevent collisions between moving trains and passing vehicles. The system uses lidar for detection. It has been installed at more than 800 level crossings on JR East and has been field-tested for about two years at a level crossing on the main line. The results show that the system is very effective in preventing falling objects. At present, China has formed a security monitoring system that integrates civil defense, physical defense, and technical defense. Advanced sensing methods have been implemented in key areas, such as power grids, cameras, lidar, millimeter-wave radar, vibration fiber optics, etc. Power grid monitoring has been deployed at road bridges over railways. However, monitoring technologies for locations such as tunnel entrances have not been systematically installed yet. Countries have designed various monitoring methods and techniques for specific risk points on railway lines. However, high-speed railways require high system security, with the characteristics of zero false alarms and low false alarms. The types of railway intrusion targets are diverse, and the climate environment is variable. How to reliably perceive and accurately identify intrusion targets under adverse weather conditions is currently a technological challenge. It is also a bottleneck that restricts the practicality of high-speed rail perimeter intrusion detection systems.

## 3. Single-Mode Identification Method for High-Speed Rail Perimeter Intrusion

### 3.1. Vision-Based Detection Method

At present, a large number of cameras are deployed around China’s railways, which can effectively reduce civil defense costs. With the development of neural networks, researchers have conducted research on perimeter intrusion detection methods based on deep learning. The University of Bremen [14] proposed a CNN-based railway obstacle intrusion detection method, which achieves improved target detection at long range. The University of Nis [15] proposed an improved edge detection method for low-quality images in railway obstacle detection. Chen [16] proposed a GA-based semi-supervised anomaly detection approach that enables the detection of foreign objects on railways without prior knowledge. Rampriya et al. [17] used methods such as faster RCNN for detection under different lighting conditions on railways and were able to achieve more than 96% accuracy. Prakash et al. [18] used the Yolov3 algorithm for forest railway track monitoring to detect large animals intruding on the track. The research reduced the accidents caused by large animal intrusion. Jiao et al. [19] used the mask R-CNN method for track identification and hazardous area segmentation to achieve fast detection of railway surface obstacles. The above methods are mainly based on algorithms developed by deep learning [20,21,22,23,24]. This type of algorithm treats the intrusion target detection problem as a regression problem. The image is represented through a neural network, and the bounding boxes and categories are predicted by dividing the image into grids. Finally, the bounding boxes, target confidence, and categories of all grids are detected to achieve end-to-end training. However, deep learning methods require a large number of training samples to effectively recognize the trained targets. However, the shapes, sizes, dimensions, and colors of foreign objects on site are diverse and cannot be exhaustively listed. Therefore, algorithms find it challenging to achieve zero false negatives.

**Figure 4:**
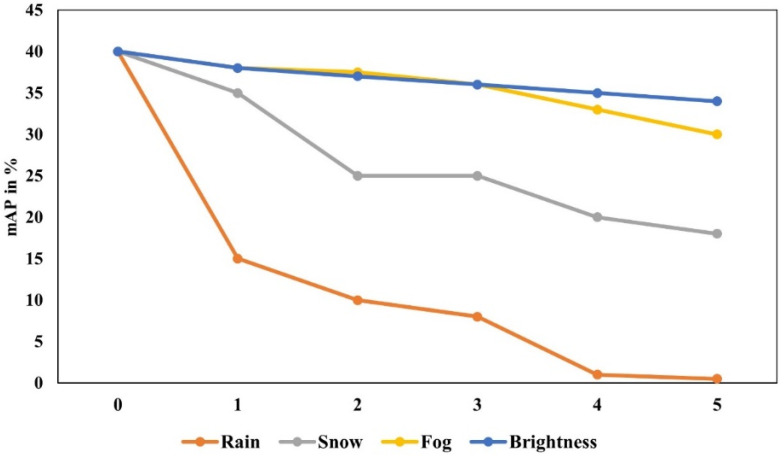
Detection accuracy of cameras under weather interference [30].

Furthermore, the meteorological conditions along the railway lines are changeable, and the terrain and environmental conditions are complex. This requires perimeter intrusion detection algorithms to have strong anti-interference capabilities, such as dealing with shaking, long distances, nighttime, and recognition interference caused by harsh weather conditions. In addressing the target recognition problem under interference conditions, Qin et al. [25] proposed a robust feature-aware network (RFA-Net) for detecting intrusion targets under complex varying conditions. They utilized integrated full-gradient distillation (IFGD) to enhance feature extraction capabilities, achieving a detection accuracy of 92.7%. Guo et al. [26] proposed a fast de-shaking method for one-dimensional grayscale projection images and a background updating algorithm, which can effectively detect intrusion targets in railway shaking scenes. Kapoor et al. [27] enhanced recognition performance under different lighting conditions by combining two-dimensional singular spectral decomposition with deep neural networks. Chen et al. [28] introduced a real-time perception and anomaly detection mechanism using deep semantic segmentation to extract real-time track areas and detect obstacles of different shapes and types based on a continuous trajectory. This method can detect small obstacles on straight tracks, curves, and turnouts and is suitable for constantly changing weather conditions. Appiah et al. [29] proposed an improved method based on YOLOV7 to enhance detection performance under adverse weather conditions such as rain and fog. Although research on adverse weather has led to performance improvements in detection, the accuracy remains below 80%. A study [30] revealed that under rain, snow, and fog interference, the camera’s detection accuracy decreased by at least 31.1%, with a maximum decrease of 60.4%. The more severe the weather interference, the more pronounced the camera’s detection effectiveness deterioration. The detection accuracies of cameras under weather interference are shown in Figure 4.

Additionally, with the emergence of large model technology, research on perimeter intrusion recognition is gradually advancing using visual-based large model technology [31]. Compared to traditional neural networks, these methods utilize a global self-attention mechanism to model semantic information in images, independent of local receptive fields. However, the timeliness of their application still requires further verification.

Although video-based intelligent monitoring technology has been widely adopted, it has to some extent reduced the monitoring burden on maintenance personnel. However, it still has shortcomings. Firstly, cameras have poor recognition accuracy in situations such as nighttime, light and shadow interference, rain, and fog. Secondly, current deep-learning methods require a large number of training samples. However, perimeter intrusion is a low-probability event, making it difficult for us to obtain a significant amount of real intrusion data.

### 3.2. Radar-Based Detection Method

Millimeter-wave radar receives electromagnetic wave signals reflected by target objects to calculate the distance, speed, and angle of the target. It has advantages such as being less affected by lighting conditions and harsh weather. Cai et al. [32] proposed a railway-level crossing obstacle detection system based on millimeter-wave radar, using signal processing algorithms to extract target signals and suppress clutter in received signals. Jing [33] addressed the issue of obstacles intruding on railway perimeters by proposing a millimeter-wave radar-based obstacle intrusion detection method, achieving identification and tracking of moving targets within the radar detection range. Yair et al. [34] utilized millimeter-wave radar with neural network architecture for reliable classification of pedestrians and animals, achieving 100% detection at close range. British scholars [35] installed radar on one side of the railway to achieve obstacle detection within a 30 m range with 0.5 m resolution, but its detection range is limited, suitable only for road intersection sections. The main reason for the short detection range of the radar is the sparse point cloud.

To address the issues of excessive noise or sparse point clouds in traditional radar recognition algorithms, many scholars have conducted related research. Pan et al. [36] proposed a motion segmentation and clustering method based on 4D millimeter-wave radar, with the addition of a motion estimation module, showcasing outstanding tracking accuracy for moving objects. To enhance the perception capability of autonomous driving, Yan et al. [37] introduced a multi-view feature network for completing 3D object detection using 4D millimeter-wave radar and incorporated a position map generation module to address the issue of insufficient feature utilization. Shi et al. [38] proposed a multi-view feature fusion network framework based on 4D millimeter-wave radar. This method can model the entire 3D scene and overcome the limitation of feature representation capacity in a sparse point cloud. While there has been extensive research on 4D millimeter-wave radar in public transportation, further exploration is needed to enhance the detection performance of 4D millimeter-wave radar in complex railway environments.

### 3.3. Lidar-Based Detection Method

Lidar has the characteristics of high detection accuracy and being unaffected by lighting conditions, making it suitable for monitoring intruding targets such as falling rocks, vegetation, and mudslides. Amaral [39] focused on obstacle detection at railway-level crossings, using the background differencing method for target identification based on lidar’s three-dimensional point cloud. Yu et al. [40] proposed a real-time track recognition method based on three-dimensional point clouds, employing a multi-scale neural network to generate predictive results for each voxel and track position. In addressing the issue of boundary monitoring for foreign object intrusion, Qu [41] first preprocesses the railway scene point cloud collected by lidar, then uses the RANSAC algorithm to partition and fit the track plane, divides the detection area based on the track position, and finally employs an improved Euclidean clustering method for obstacle classification. Wisultschew [42] used 3D lidar for real-time target detection and tracking in railway-level crossing scenes. Shinoda et al. [43] focused on scenarios within tunnels, using lidar for target detection of personnel and vehicles. However, the above-mentioned literature primarily employed background differencing and clustering methods. With the increasing application of deep learning methods in the field of point cloud object detection, numerous deep learning-based approaches are gradually being developed and researched, such as PointNet [44], VoxelNet [45], PointPillars [46], and PointRCNN [47], among others. Deep learning methods offer powerful learning capabilities and high recognition accuracy. However, they require a large amount of sample data for network training and optimization. This leads to algorithms being time-consuming. Despite the extensive research on lidar recognition methods, there are still challenges, such as susceptibility to rain, fog, and other disturbances, when applied on a large scale in the railway sector.

### 3.4. Fiber-Based Detection Method

Fiber optic sensing technology utilizes changes in the characteristic parameters of light propagating through the fiber caused by external vibrations for measurement and data transmission, possessing features such as high sensitivity and strong interference resistance. Sinha et al. [48] employed fiber optic sensors to detect rocks and wood of different weights falling at different distances on railheads, ballasts, and sleepers, enabling the detection of rocks and wood falling on railway tracks within a distance of 500 m. Catalano et al. [49] used fiber optic grating sensors to monitor perimeter scenes for unauthorized personnel intrusion. Nan et al. [50] proposed a distributed vibration fiber optic monitoring method, utilizing ultra-weak fiber Bragg grating sensor technology to collect the distributed vibration responses of moving trains and intruders, effectively tracking trains and monitoring personnel intrusion. Qu et al. [51] proposed a fiber optic sensing technology based on phase-sensitive detection to capture the raw vibration signal of railway tracks over a period of time. This method involves filtering, short-time Fourier transform, and autoregressive model spectrum estimation. After analyzing the frequency signal characteristics, it can determine whether a perimeter intrusion event has occurred on the track. Catalano [52], based on integrated vibration fiber optics, developed a method for detecting railway perimeter personnel intrusion events using fiber Bragg gratings. Fang [53] employed an asymmetric dual Mach–Zehnder interferometric fiber optic sensing technology utilizing the RBF neural network and an improved wavelet packet denoising method for high-speed railway perimeter intrusion detection. Vibration fiber optics exhibit high sensitivity suitable for long-distance detection but have poor robustness, making them prone to false alarms.

### 3.5. Infrared-Based Detection Method

To address the issue of poor camera recognition in dark conditions, a method for identifying railway obstacles based on monocular thermal imaging was proposed by Swedish scholars [54]. Indonesian railways [55] also use infrared thermal imaging to detect obstacles in front of trains and signal locomotive drivers to make decisions to reduce speed or stop the train through indicator lights. Kapoor [56] introduced a new method using thermal imaging to identify objects (obstacles) on railway tracks in front of moving trains, with results showing an accuracy rate of approximately 83%. Wang [57] presented a multi-feature fusion and attention-enhanced anchor-free foreign object detection algorithm, as well as a feature-enhanced railway foreign object tracking detection algorithm. Li et al. [58] proposed a method for railway foreign object detection, first synthesizing infrared railway images using generative adversarial networks and then predicting synthetic infrared foreign object targets using a single-shot multi-box model. However, this method suffers from poor real-time performance due to multiple stages of generation and detection. Yang et al. [59] put forward a cascaded enhanced single-point multi-box detection SSD algorithm, enhancing the extraction of infrared target information through a cascade R-CNN feature network and combining it with a tracking network for mobile target tracking detection. Infrared thermal imaging cameras have good detection performance at night. They can identify targets hidden behind vegetation, with a wide visual range, and are less susceptible to environmental interference. However, the drawback of infrared technology is its inability to recognize objects with small temperature differences in the environment and its incapacity to identify detailed features of invading targets.

### 3.6. Summarize

Summarizing the advantages and disadvantages of the above sensor detection methods, the results are shown in Table 1.

## 4. Multi-Sensor Identification Method for High-Speed Rail Perimeter Intrusion

**Figure 5:**
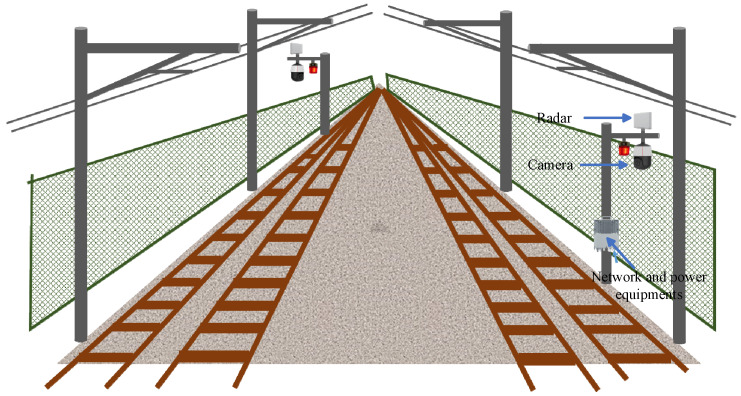
The ground installation method.

Due to the current limitations of single sensors in monitoring, it is necessary to combine the technological advantages of multiple sensors and conduct research on multi-sensor fusion detection technology. Currently, multi-sensor fusion monitoring devices include two deployment methods: onboard installation and ground installation. Considering that the braking distance of high-speed trains exceeds 2 km, it is not possible for existing onboard monitoring devices to effectively detect targets beyond this range. Therefore, we believe that ground installation is the optimal solution to address this issue. Using radar as an example, the ground installation method is shown in Figure 5.

In addition, multi-sensor fusion algorithms can be divided into three levels based on different data processing methods: data-level fusion, feature-level fusion, and decision-level fusion. Data-level fusion involves aggregating and fusing various data collected by different sensors, then analyzing and identifying the fused data. The advantage of this method is to ensure the richest and most accurate information from the original data as possible. However, the drawbacks are that the scale of the original data is too large, the data formats from different sensors are not completely consistent, the fusion processing is complex, and real-time performance is relatively low. Feature-level fusion involves extracting features from the data collected by different sensors, merging the extracted feature information, and analyzing and identifying the fused features. This method reduces the bandwidth requirements for data transmission and improves some aspects of real-time performance.

Decision-level fusion involves merging the results output by different sensors separately. This fusion method, according to application requirements, achieves analysis and judgment by establishing fusion rules. The advantage of this approach is its strong real-time performance, good noise resistance, and fault tolerance. However, it suffers from significant data information loss, and its accuracy heavily relies on the results of the preceding processing stages. Combining the characteristics of multiple sensors such as cameras, millimeter-wave radar, laser radar, vibration fiber optics, and infrared thermal imaging has become a hot research topic for achieving precise identification of multi-modal fusion data. Combining the characteristics of multiple sensors such as cameras, radar, lidar, fiber, and infrared, achieving precise detection of multi-sensor fusion data has become a hot research topic.

### 4.1. Methods Based on Radar and Camera Fusion

Atsutake et al. [60] proposed a millimeter-wave YOLO architecture, achieving high-precision object classification and position recognition by applying different detectors to each set of distance data. Liu et al. [61] introduced a traceless Kalman filtering method for the fusion of millimeter-wave radar and cameras, used to track detected targets in the image plane and monitor new target objects in the scene. Wang et al. [62] presented a robust target detection and classification algorithm based on the fusion of millimeter-wave (MMW) radar and cameras, with an accuracy 89.42% higher than traditional radar signal algorithms and 32.76% higher than faster R-CNN, especially in low-light and strong electromagnetic interference environments. Lamane et al. [63] utilized FMCW radar and visual fusion identification for target detection and classification, achieving higher accuracy compared to existing methods. Song et al. [64] proposed a multi-source deep learning target detection network based on the fusion of millimeter-wave radar and vision for detection, effectively reducing missed detection rates under insufficient lighting conditions. With the maturity of 4D millimeter-wave radar technology, Guan et al. [65] introduced a fusion model of vision and 4D millimeter-wave radar, employing a panoramic perception training strategy based on uncertainty to achieve better performance in harsh weather and poor lighting conditions. Shuai et al. [66] studied feature-level fusion methods for millimeter-wave radar and cameras, significantly enhancing detection capabilities in low-light scenes. The team from the China Academy of Railway Sciences [67] has proposed a radar–video fusion detection method for identifying and tracking perimeter intrusions by personnel on high-speed rails, enabling round-the-clock monitoring. Ding et al. [68] focused on target detection in snowy conditions and introduced a feature fusion module to construct a lightweight object detection network, addressing challenges such as blurriness, distortion, and snow coverage. Albert et al. [69] proposed a method for integrating camera and radar data. This approach involves establishing a recurrent neural network to process the time series of object positions and speeds. Centerfusion [70] is a feature fusion method for three-dimensional object detection using radar and camera data. Initially, a center point detection network is used to detect objects by recognizing the center points on the image. Then, a novel method based on a frustum is employed to address key data association issues, associating radar detections with the corresponding object center points. Compared to state-of-the-art vision-based algorithms, the detection accuracy has been improved by over 12%.

Research has shown that the fusion of cameras and millimeter-wave radars has a significant impact on mobile target detection. However, there is still a need to improve the recognition of static foreign objects and enhance the ability to fuse 4D radar information, which can further improve the performance of detection methods.

### 4.2. Methods Based on Lidar and Camera Fusion

Previous research has designed various methods for the fusion of cameras and lidar in perimeter intrusion detection. Han proposed a fusion method for lidar and cameras [71], which projects lidar point clouds onto images through cross-calibration to obtain sparse depth images, followed by classification and recognition. Another method involves using image detection algorithms to generate a series of bounding boxes, based on which a series of 3D frustums are created and projected into a 3D point cloud space for recognition [72]. Wang et al. [73] proposed a multi-sensor framework that integrates camera and lidar data, using a segmentation + decision-level fusion method for monitoring obstacles on railway tracks. Yu et al. [74] introduced a railway foreign object detection system based on the combination of lidar and video, constructing three-dimensional point cloud data and performing denoising, track segmentation, and target clustering, effectively reconstructing the track’s topological structure. Miickel [75] presented a fusion detection method for cameras and lidars, achieving railway obstacle detection with a range of up to 400 m under typical working conditions. Shen et al. [76] proposed a multi-source object detection network that integrates target tracking based on camera and lidar fusion (YCANet). This method utilizes an improved YOLOv7 and CenterPoint for image and point cloud detection, respectively, and employs aggregated Euclidean distance (AED) as a new metric in the data association module for tracking results of both images and point clouds. Xiao et al. [77] used multi-sensor technology based on computer vision and lidar for real-time collection of video images and ranging data in the track area, followed by preprocessing of collected videos and delineation of regions of interest. Subsequently, obstacle detection was performed in the regions of interest to obtain the geometric features and location information of obstacles. Finally, based on the severity of the obstacles, the impact on train operation was determined, and the detection results were transmitted to the corresponding trains. Wen et al. [78] designed a strategy for obstacle detection in complex weather conditions by integrating cameras and lidar and proposed a multimodal contrastive learning method. In extreme and harsh weather conditions such as rain, snow, and fog, Mai et al. [79] proposed a lidar and camera stereo fusion network to address the issue of data distortion, significantly improving 3D detection performance in foggy weather conditions. Our team [80] conducted research on tunnel entrance scenes and studied a fusion monitoring scheme using lidar and cameras capable of detecting foreign objects of 20 cm^3^ within a range of 60 m under heavy rain and similar weather conditions. Although both domestic and international research institutions have conducted studies on the fusion of lidar and video technologies [81], the application of lidar and video fusion methods in railways still faces the following challenges: (1) The detection distance of lidar and camera fusion is limited, leading to unclear point clouds and images at long distances; (2) Lidar and cameras are unable to function properly under adverse weather conditions (such as heavy rain and thick fog); (3) The cost-effectiveness of lidar and camera fusion technology is also a significant issue that cannot be overlooked.

### 4.3. Methods Based on Infrared and Camera Fusion

Visible light images are suitable for the human visual perception system, with high resolution and rich detail features. Infrared images depict the thermal radiation of objects, which can resist interference from harsh environments, but they typically have low image resolution. The fusion of images from these two modalities can provide images with high contrast and rich texture details while resisting interference from harsh environments, which is beneficial for subsequent target detection. Gasparini et al. [82] proposed a method for detecting intrusions in front of trains at night based on the fusion of visible and infrared thermal images, using deep learning techniques for automatic detection. Zhou [83] presented a railway foreign object intrusion detection method based on multi-source image fusion, improving registration algorithms based on SURF feature points. The decision-level fusion of infrared and visible light images compensates for the deficiencies in detection under shadows and nighttime conditions in visible light images. Xu [84] improved the ViBe detection algorithm and proposed a method for enhancing target fusion in railway foreign object intrusion detection, addressing the issue of single visible light image recognition failure in nighttime and adverse weather conditions. Italian scholars [85] installed infrared thermal imaging and cameras in front of trains to fuse visible light images and thermal images for detecting obstacles ahead on the railway. They introduced a deep learning framework with two sequential modules, an autoencoder network and a classification recognition network, and collected camera and thermal imaging data in railway scenarios. In other fields, Kim et al. [86] presented a method for detecting and tracking sea-level targets using target detection and robust statistics based on image and infrared sensors, which are robust against sensor vibration and occlusion. Xie et al. [87] proposed a local feature descriptor for the registration of visible light and infrared images, encoding anisotropic features of multiscale edges and texture information to achieve better detection results. Currently, there is limited research on the fusion recognition of infrared thermal imaging and visible light images in the railway field. The main reason is that it is difficult to distinguish between the temperature of the intrusion target and the environmental temperature when the temperature difference is small. Therefore, we still need to improve the detection accuracy in scenarios with low-temperature differences.

### 4.4. Methods Based on Fiber and Camera Fusion

The fusion monitoring area of vibrating optical fiber and video is mainly in the boundary area of railway lines. Zhou [88] proposed a railway perimeter monitoring system based on vibrating optical fiber and video analysis technology. When the vibrating optical fiber detects an intrusion, the video will be called to retrieve the video information at the intrusion location and analyze it. This system can meet the long-distance and large-scale monitoring requirements of railways. Bai et al. [89] proposed a perimeter protection system combining vibrating optical fiber and video analysis technology suitable for high-speed railway lines. This system uses vibrating optical fiber to achieve precise positioning of intrusion events and reviews intrusion events through video analysis technology. Li et al. [90] proposed a high-speed rail perimeter intrusion perception and identification system that integrates distributed optical fiber sensing technology, video linkage technology, and intelligent recognition technology. This system uses distributed vibrating optical fiber to identify intrusion locations and collects on-site data for model training, reducing data processing dimensions and false alarm rates. The China National Railway Group [91] integrated pre-processed vibrating optical fiber data with video data for decision fusion, enhancing monitoring accuracy under severe weather conditions. Ma [92] designed a distributed optical fiber intrusion behavior monitoring system based on faster R-CNN. This system uses the network for feature processing of optical fiber data and analyzes videos using a human skeleton extraction model based on ST-GCN. By correlating the two types of data, the system can accurately monitor four different intrusion behaviors. However, the monitoring method based on the fusion of fibers and cameras is primarily contact-based monitoring. Fibers cannot provide three-dimensional protection, leading to monitoring blind spots and gaps, making them only suitable for boundary areas within the perimeter range.

### 4.5. Other Methods

With the rapid development of sensor technology, an increasing number of integrated monitoring methods are being applied in the railway field. Zhao [93] used the fusion of radar and lidar to preliminarily achieve obstacle detection within the track limits. Garcia et al. [94] proposed a fusion system using infrared and ultrasonic sensors to detect targets on railway tracks and employed principal component analysis for data extraction. The Railway Technical Research Institute of Japan [95] proposed a method of detecting obstacles on railways using the fusion of millimeter-wave radar and vibrating optical fibers. The proposed method can detect people and non-metallic objects entering the track area up to approximately 200 m from the installation point. Indian Railways [96] has proposed an integrated system for detecting obstacles on tracks under fog, smoke, and heavy rain conditions. This system utilizes cameras, millimeter-wave radar, laser radar, and infrared thermal imaging. The cameras capture long-distance images of the track, displaying them in real-time on a mini screen fixed in the locomotive cabin. Short-range, mid-range, and long-range combined millimeter-wave radar sensor systems are used for continuous detection of obstacles in the locomotive driver’s blind spots on the track. Long-range laser and infrared illuminators are employed for monitoring in zero visibility conditions. In addition, Chen et al. have researched a general fusion framework for autonomous driving using radar, lidar, and cameras [97], achieving the best object detection performance on a publicly available multi-source data fusion dataset. Ricardo et al. have developed a perception architecture based on an evidence framework that fuses radar, lidar, and cameras to address the detection and tracking of pedestrians, cyclists, cars, and trucks [98]. Mario has proposed a method for multimodal recognition under adverse weather conditions, combining lidar, radar, cameras, and infrared thermal imaging, introducing a deep fusion network for robust fusion that incorporates an entropy-based approach and adaptive fusion features [99]. Although various fusion methods have been proposed, the applicable boundaries of the above methods are still unclear.

## 5. High-Speed Rail Perimeter Intrusion Multi-Sensor Data

### 5.1. Multi-Sensor Data Alignment Method

In order to realize the effective fusion of multi-sensor data, data alignment between various sensors is a prerequisite, including for internal and external parameter calibration. The internal parameter calibration is an inherent property of the camera that realizes the projection of the target in the camera coordinate system to the imaging plane, including the camera matrix and the distortion coefficient. These parameters are determined by calculating the relationship between feature points with known coordinates on the calibration object and their corresponding pixel points on the image. A popular method for calibrating the internal reference is the Zhang Zhengyou calibration method. The external parameter is the mapping matrix between two sensor data points. By calculating the mapping matrix, we can transform the multi-sensor data in the system to be used together under the same reference coordinate system. This is a key step in multi-sensor data fusion.

In the alignment method for point cloud and image data, extrinsic parameter calibration realizes the projection of the target in the lidar/radar coordinate system to the camera coordinate system. The extrinsic parameters of radars and cameras consist of rotation and translation matrices. At present, the external parameter calibration method can be divided into two categories [100,101]: direct inverse solution and the learning-based method.

The direct inverse solution method utilizes the feature points of the calibration plate for calibration. The learning-based method achieves calibration by directly extracting and automatically matching structural features in the environment. In terms of data alignment methods for radar and images, Wang et al. [102] proposed a high-precision calibration method based on the region of interest (ROI) and artificial potential field. Among the alignment methods for images and infrared, image feature-based alignment [103] and deep learning-based alignment methods [104,105] were proposed. Learning-based methods can avoid image shifts and distortions caused by external reasons such as jitter in the imaging platform. This can improve the robustness of detection.

### 5.2. Railway Scene Multi-Sensor Dataset

**Figure 6:**
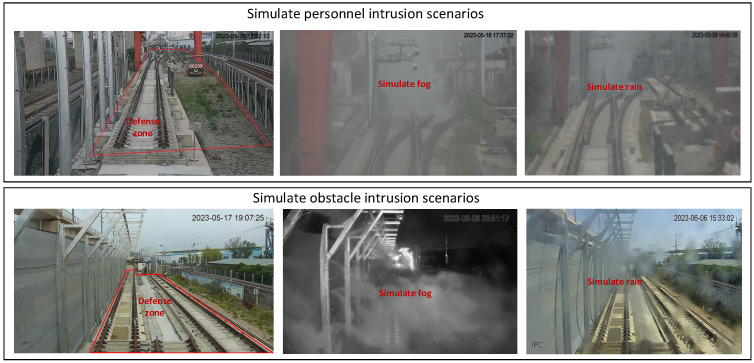
Simulation of intrusion scenarios [67].

Multi-sensor fusion relies heavily on pre-training data. A large amount of manually labeled scene intrusion data can drive the algorithm to achieve good performance. Because railroad perimeter intrusion is a low-probability event and it is more likely to happen during severe weather conditions like strong winds, heavy rain, snowfall, and thick fog, it was difficult for us to collect intrusion multi-sensor datasets in railway scenarios. To solve this problem, Gianluca [106] proposed TrainSim, which recreates realistic railway scenes in a virtual scenario. This study automatically generated training datasets from simulated lidar and cameras. Wu [107] proposed an automatic multi-sensor joint calibration method and constructed a point cloud dataset for a railway scene. To realize the automatic driving of a train, RailSem19 [108] collected video clips of railway scenes from 38 countries/regions with different weather, lighting, and seasons in the train perspectives. This dataset consists of 8500 annotated short sequences from the ego-perspective of trains, including over 1000 examples with railway crossings and 1200 tram scenes. This dataset displayed images of trains, turnouts, platforms, buffer stations, railway signs, and railway signals. Deutsche Bahn produced a multi-sensor dataset, OSDaR23 [109], which includes lidar, millimeter-wave radar, visible light camera, and red and infrared camera data and labels 200,000 various object categories related to the railway environment. For the case of installing the sensors on the ground, we [67] proposed an acquisition scheme of mounting the sensors on poles. A realistic railway intrusion scenario was built, and multi-sensor fusion data was collected using real intrusions. The scenario is shown in Figure 6.

Since the environment of railways is complex and changeable, sensor combinations and recognition methods are diverse, which requires a wide range of data sets for training and evaluation. Therefore, multi-sensor fusion data sets of high-speed railway perimeter intrusion scenes still face multi-scene, multi-day, multi-behavior, and multi-species characteristics.

## 6. Risk Assessment of Railway Safety

To apply the results of perimeter intrusion detection to the high-speed railway system, it is necessary to conduct research on perimeter intrusion risk assessment methods. Risk assessment can provide accurate data references for railway inspectors, as well as objective decision-making support for management departments. For the safety of passengers at railway stations, the Laboratory for Track Engineering and Operations for Future Uncertainties [110,111,112] uses the decision tree (DT) method for safety classification and analysis. Additionally, adaptive neuro-fuzzy inference is used to assess the congestion level of train stations. A risk decision-making framework based on probabilistic risk assessment (PRA) has also been proposed. Aiming at the risk of shunting on dedicated lines, Zhang et al. [113] proposed a risk assessment method based on fuzzy reasoning. The Czech Republic [114] adopts a fuzzy mixed method to assess the risks of railway infrastructure. Liu et al. [115] proposed an ensemble learning algorithm for railway signal safety and constructed a comprehensive risk assessment method. In response to safety issues along railway lines, the China National Railway Group [116,117,118] has classified risks into four levels and developed railway safety risk control measures and evaluation methods. However, the above methods are mainly based on manual evaluation data, and data-driven evaluation methods require further research. In addition, information including target types and intrusion locations based on perimeter intrusion has not been effectively applied in evaluation methods and disposal measures.

## 7. Conclusions

To our knowledge, this article provides a comprehensive overview of perimeter intrusion detection in high-speed railways for the first time, including single-sensor detection and multi-sensor fusion detection. Compared to single-sensor detection, the use of multiple sensors can enhance the accuracy of perimeter intrusion detection. Through simulated environment testing, we can gain the application boundaries of multi-sensor technology. However, under adverse conditions, multi-sensor detection methods still face performance degradation. The main reason is that when the wavelength of electromagnetic waves emitted or received by the above sensors is smaller than the diameter of the rain and fog particles, the electromagnetic waves cannot penetrate or diffract through the rain and fog particles. However, adverse weather conditions are inevitable. Only by overcoming these challenges can multi-sensor fusion technology be further applied. Compared to cameras, infrared, and lidar, it can be said that radar has greater potential. In addition, another challenge currently faced is the lack of specific datasets in the railway domain, especially intrusion data under adverse weather conditions. Therefore, for railway applications, we need datasets that cover full railway scenarios to conduct rigorous evaluations of existing methods. Moreover, how to integrate the detection results of the existing methods into the current railway system still requires further exploration.

The safety level of high-speed railways continues to improve. In this study, we focus on the recent fusion strategies for multiple sensors in the railway field. In Section 3, we discussed single-sensor recognition algorithms in detail. We found that the performance of single sensors is limited in low light or bad weather conditions. Therefore, by analyzing these drawbacks, it becomes evident that it is necessary to fuse multi-sensor data. In Section 4, we discussed multi-sensor fusion strategies from recent years’ research and analyzed the problems of recognition methods based on multi-sensor fusion. Furthermore, we provided a necessary summary of multi-sensor fusion data alignment and datasets. However, we found that there are still some shortcomings in the multi-sensor fusion method through this survey. Therefore, we propose future research perspectives.

(1) Reliable perception under severe weather conditions

Currently, most of the existing research focuses on the accurate classification and identification of targets. Accurate sensing of perimeter intrusion targets by sensors has been neglected. As a result, the adaptability and stability of sensors in adverse weather conditions are reduced. For example, camera images are blurred in dense fog conditions and plagued by interfering light spots in heavy rain conditions. Lidar point clouds also become less in dense fog conditions, while radar detection distances become shorter in heavy rain conditions. The result is that the sensor cannot detect the target behind rain and fog. Therefore, the research and innovation of wide-wavelength and high-resolution sensor technology should be increased to realize all-weather sensing of intrusion targets.

(2) Accurate recognition using multi-sensor fusion data

In recent years, more and more research has focused on deep learning methods and multi-sensor fusion methods to improve the reliability of the railway system. Existing recognition methods can accomplish perimeter intrusion monitoring better under sunny weather conditions. When the acquired monitoring data are unstable, incomplete, and inaccurate, the recognition performance drops dramatically. Therefore, the research on multi-sensor fusion recognition algorithms in railway scenarios should be deepened, and the effective information of multi-sensor fusion data should be deeply excavated. This can improve the performance and adaptability of the railway environmental safety monitoring system and provide strong protection for trains.

The railway system requires high accuracy in perimeter intrusion detection. To be useful in the railway application, the existing sensing technology and artificial intelligence technology still need to be improved.

## Figures and Tables

**Table 1:** Pros/cons of various sensor detection methods.

Sensor	Pros	Cons
Video	Strong recognition ability. Easy to deploy and maintain.	Poor generalization capabilities for intrusion targets. Poor adaptability to the weather.
Radar	Strong adaptability to the environment. Long detection distance for moving objects.	Point clouds are sparse and data processing is difficult.
Lidar	High ranging accuracy. Not affected by light.	Fog weather recognition performance decreases. Costs are higher.
Fiber	Low false negative rate. Strong adaptability to the environment.	High false alarm rate. Poor positioning accuracy.
Infrared	Good nighttime detection performance. Large viewing range.	Poor low temperature differential detection. Unable to identify detailed features.

## Data Availability

Not applicable.
